# Impact of Telemedicine Versus In-Person Pediatric Outpatient Type 1 Diabetes Visits on Immediate Glycemic Control: Retrospective Chart Review

**DOI:** 10.2196/58579

**Published:** 2024-10-01

**Authors:** Christopher Ferber, Steven D Mittelman, Tannaz Moin, Holly Wilhalme, Rebecca Hicks

**Affiliations:** 1 Division of Pediatric Endocrinology University of California Los Angeles Los Angeles, CA United States; 2 Department of Pediatrics Endocrine and Diabetes Center Miller Children's and Women's Hospital Long Beach Long Beach, CA United States; 3 Children's Discovery and Innovation Institute UCLA Mattel Children's Hospital David Geffen School of Medicine Los Angeles, CA United States; 4 Division of Endocrinology, Diabetes, and Metabolism David Geffen School of Medicine at UCLA Los Angeles, CA United States; 5 HSR&D Center for the Study of Healthcare Innovation, Implementation & Policy VA Greater Los Angeles Healthcare System Los Angeles, CA United States; 6 Department of Medicine Statistics Core Division of General Internal Medicine and Health Services Research University of California Los Angeles Los Angeles, CA United States

**Keywords:** diabetes, type 1 diabetes, pediatrics, continuous glucose monitoring, time in range, glucose management indicator, telemedicine, screening labs

## Abstract

**Background:**

Children and adolescents with type 1 diabetes require frequent outpatient evaluation to assess glucose trends, modify insulin doses, and screen for comorbidities. Continuous glucose monitoring (CGM) provides a detailed glycemic control assessment. Telemedicine has been increasingly used since the COVID-19 pandemic.

**Objective:**

To investigate CGM profile parameter improvement immediately following pediatric outpatient diabetes visits and determine if visit modality impacted these metrics, completion of screening laboratory tests, or diabetic emergency occurrence.

**Methods:**

A dual-center retrospective review of medical records assessed the CGM metrics time in range and glucose management indicator for pediatric outpatient diabetes visits during 2021. Baseline values were compared with those at 2 and 4 weeks post visit. Rates of completion of screening laboratory tests and diabetic emergencies following visits were determined.

**Results:**

A total of 269 outpatient visits (41.2% telemedicine) were included. Mean time in range increased by 1.63% and 1.35% at 2 and 4 weeks post visit (*P*=.003 and .01, respectively). Mean glucose management indicator decreased by 0.07% and 0.06% at 2 and 4 weeks post visit (*P*=.003 and .02, respectively). These improvements in time in range and glucose management indicator were seen across both telemedicine visits and in-person visits without a significant difference. However, patients seen in person were 2.69 times more likely to complete screening laboratory tests (*P*=.03). Diabetic emergencies occurred too infrequently to analyze.

**Conclusions:**

Our findings demonstrate an immediate improvement in CGM metrics following outpatient visits, regardless of modality. While statistically significant, the magnitude of these changes was small; hence, multiple visits over time would be required to achieve clinically relevant improvement. However, completion of screening laboratory tests was found to be more likely after visits occurring in person. Therefore, we suggest a hybrid approach that allows patient convenience with telemedicine but also incorporates periodic in-person assessment.

## Introduction

Type 1 diabetes affects millions worldwide with up to 75% of cases diagnosed during childhood [1]. Pediatric type 1 diabetes care necessitates frequent outpatient visits to assess glycemic trends and adjust insulin doses, stay up to date with routine screening [2,3], provide reinforcement of diabetes education such as management of hypoglycemia, hyperglycemia, and illnesses, and prevent potentially life-threatening diabetic emergencies including level 3 hypoglycemia or diabetic ketoacidosis. In the past decade, continuous glucose monitors (CGMs) have become increasingly prevalent in place of traditional finger sticks [4]. By providing more frequent real-time blood glucose data with trends and safety alerts for glycemic excursions, CGM use has positive effects on clinical diabetes outcomes including less frequent episodes of hypoglycemia and lower hemoglobin A_1c_ (HbA_1c_) values [5,6]. Time in range (TIR) and glucose management indicator (GMI) are 2 key measures of glucose control generated from CGM data. TIR is the percentage of the time during a CGM window for which the blood sugar falls within a preset target range (typically 70-180 mg/dL). GMI is a surrogate measure for HbA_1c_ calculated from all the available blood sugar data used to quantify overall glycemic control. GMI has the advantage of being applicable to custom time periods as opposed to the fixed red blood cell lifespan of 90-120 days, which HbA_1c_ reflects. Frequent monitoring of HbA_1c_ remains the gold standard of assessing diabetes management with tighter glycemic control being associated with less frequent complications of diabetes over time, even in youth [7-9]. But with CGM use rising, many have advocated for using these CGM based metrics to assess overall diabetes control in addition to, or even in lieu of, HbA_1c_ [10,11]. In addition to glycemic control monitoring, outpatient type 1 diabetes visits also serve the purpose of making sure patients are up to date with routine screening. The American Diabetes Association recommends periodic laboratory assessment to screen for comorbidities of type 1 diabetes, as well as associated autoimmune diseases [2,3,12,13]. Finally, these visits reiterate diabetes education to patients and families which can help prevent complications of the disease including severe hypoglycemia, or the life-threatening diabetic ketoacidosis that can result from untreated hyperglycemia.

The use of telemedicine for outpatient medical visits has skyrocketed in recent years as a response to the COVID-19 pandemic [14]. Outpatient type 1 diabetes care was no exception, with some centers citing as much as 99% of their visits being converted to telemedicine within the first year of the pandemic [15]. However, even before the pandemic, telemedicine had been studied as a possible modality for type 1 diabetes health care delivery given the ability to evaluate objective data from glucose and CGM logs to guide management decisions. Telemedicine visits have been shown to improve diabetes clinical outcomes [16], while potentially removing key barriers, such as geographic distance, for patients and families. Even after widespread vaccination strategies allowed the safe return of in-person visits, it has been reported that many patients and families continue to express a preference for telemedicine [17].

Because clinic visits generally entail adjusting insulin doses, promoting adherence, and reinforcing education about insulin administration and carbohydrate counting, it is reasonable to expect that CGM profile parameters should improve immediately after an outpatient encounter. Previous studies have confirmed that increased outpatient type 1 diabetes visits lead to improved clinical outcomes [18,19]. To our knowledge, our study is the first to evaluate emerging CGM profile parameters in pediatric patients in the periods directly before and after outpatient follow-up visits, and concurrently examine whether telemedicine versus in-person visits have differential effects on glycemic control, completion of screening laboratory tests, or frequency of diabetic emergencies.

## Methods

### Overview

Our study was conducted across 2 diverse pediatric endocrinology sites in Southern California, University of California Los Angeles Mattel Children’s Hospital (UCLA) in Westwood and Memorial Care Miller Children’s and Women’s Hospital (MCH) in Long Beach. Electronic health record (EHR) data were retrospectively collected for pediatric patients with type 1 diabetes (based on the *ICD-10* [*International Statistical Classification of Diseases, Tenth Revision*] codes at visits) using Dexcom CGM who presented for at least 1 outpatient follow-up appointment during the study window of calendar year 2021. We included visits with patients who were aged 5-21 years old with disease duration at least 1-year, last HbA_1c_ <10%, a minimum of 70% CGM use, and no serious and possibly confounding medical conditions. We excluded patients that were documented to have type 2 diabetes or indeterminate type.

Baseline demographic information collected from the EHR included patient age, sex, race, ethnicity, duration of disease, insurance type, and insulin pump use. For each visit, CGM TIR and GMI were collected from the Dexcom Clarity app at 4 different time points, that is, 4 weeks and 2 weeks before each visit and then 2 weeks and 4 weeks afterward. Notably, the parameters for each 4-week period were inclusive of the corresponding 2-week period. Changes from baseline analyses were examined comparing TIR and GMI values from 4 weeks before the visit with the values obtained at 2 and 4 weeks post visit. Because of this design, analyses were performed at the visit level to look for changes in these parameters to account for patients presenting for multiple visits during the study window. Completion of screening laboratory tests completion was defined as a binary outcome and considered “complete” if all recommended laboratory tests were up to date by the next outpatient visit. EHR data were also collected on all diabetic emergencies following each visit during the study window, defined as a binary yes/no for the occurrence of glucagon use or a diabetes-related emergency department visits or hospitalization occurring before the next outpatient visit.

### Ethical Considerations

Institutional review board approval was obtained at both UCLA (IRB#21-002033) and MCH (IRB #278-22). Patient consent was waived given retrospective chart review study design.

### Statistical Methods

Patient characteristics were summarized overall and by site using means, SDs, medians, and IQRs for continuous variables, and frequencies and percentages for categorical variables. The number of visits per patient were also summarized overall and by visit type (in-person vs telemedicine). Random effects models were used for all statistical analyses since multiple visits could be potentially included for each patient. First, to examine differences in TIR and GMI over time, we conducted a linear mixed effects model using data from both sites with fixed effects for time (2 weeks and 4 weeks) and the baseline value (4 weeks before the visit). We then compared changes in TIR and GMI from baseline (4 weeks before the visit) with 2 and 4 weeks post visit by visit modality using a linear mixed effects model with fixed effects for visit type (ie, telemedicine vs in-person visit) and the baseline measurement. A second model added an interaction term between time and visit type to determine if any changes by visit type differed by time point. Multivariate models were also constructed to assess the effect of potential confounding variables collected such as age, sex, site, duration of disease, race, ethnicity, insurance type, and pump use that could affect measured outcomes. A mixed effects logistic regression model was used to determine if there was an association between visit type and the odds of completing screening laboratory tests. All analyses were conducted in SAS (version 9.4; SAS Institute). *P* values of <.05 were considered statistically significant.

## Results

**Figure 1:**
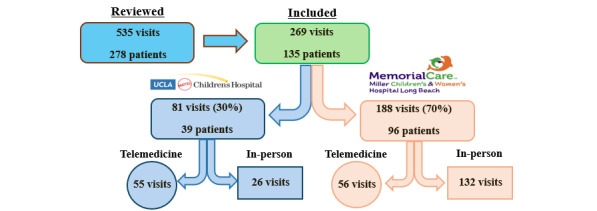
Visit breakdown.

A total of 535 visits across 278 patients at both sites were considered, of which 269 outpatient visits met inclusion criteria among 135 unique patients. Of these, 81 visits among 39 patients took place at UCLA and the remaining 188 visits among 96 patients were at MCH. Of all included visits, 111 were performed by telemedicine (41.2%, Figure 1).

A total of 73 patients within the study were male (n=73, 54.1%), mean age was 13.3 years old, and mean duration of type 1 diabetes was 5 years. There were no significant differences in these metrics between sites. However, insurance type, race, and ethnicity varied significantly between UCLA and MCH. Of the patients seen at UCLA (36/39, 92.3%) had private insurance compared with only (43/96, 44.8%) of patients seen at MCH. A majority of patients seen at UCLA were White (29/39, 74.4%), followed by Asian (2/39, 5.1%) with (7/39, 17.9%) documented as “Other” or “Unknown” and (1/39, 2.6%) with Hispanic or Latino ethnicity. Conversely, Hispanic or Latino ethnicity comprised (57/96, 59.4%) of patients seen at MCH, followed by White at (25/96, 26%), Black at (7/96, 7.3%), and (10/96, 10.4%) documented as “Other” or “Unknown.” Finally, most patients across both sites used an insulin pump, encompassing (23/39, 59%) of UCLA patients and (52/96, 54.2%) of MCH patients (Table 1).

**Figure 2:**
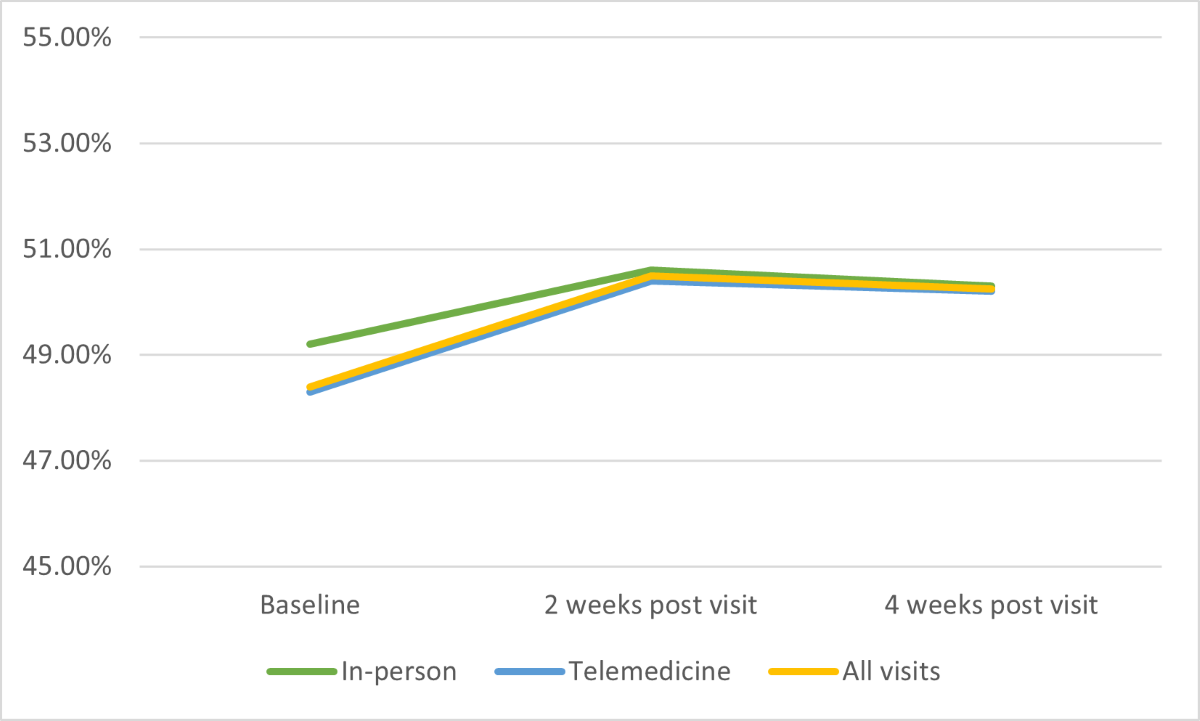
Time in range.

**Figure 3:**
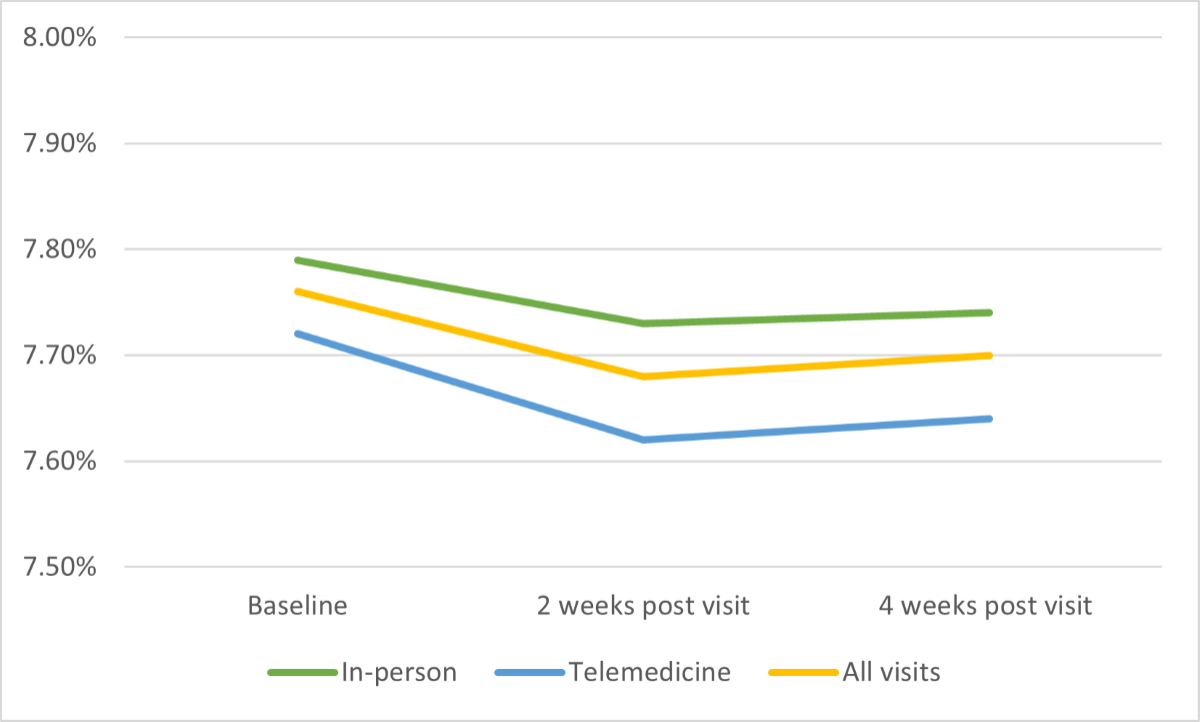
Glucose management indicator.

Across all visits, mean TIR increased by 1.63% and 1.35% during the 2-week and 4-week period after each visit, respectively (*P*=.003 and *P*=.01, respectively; Figure 2). Mean GMI decreased by 0.07% and 0.06% during the 2-week and 4-week period after each visit, respectively (*P*=.003 and *P*=.02, respectively; Figure 3). TIR and GMI at 2 and 4 weeks post visit were not statistically different from each other (*P*=.61 and *P*=.51, respectively). Following telemedicine visits, TIR increased from baseline by 1.9% at 2 weeks and 1.7% at 4 weeks, while those seen in-person had TIR improvement from baseline of 1.4% at 2 weeks and 1.1% at 4 weeks. This change in TIR between visit modality was not significant at either time point (*P*=.54 and *P*=.48, respectively; Figure 2). GMI following telemedicine visits decreased from baseline by 0.1% at 2 weeks and 0.08% at 4 weeks, while those seen in-person had GMI decrease from baseline by 0.06% at 2 weeks and 0.05% at 4 weeks. This change in GMI between visit modality was also not significant at either time point (*P*=.39 and *P*=.44, respectively; Figure 3). After adjusting for site, age, sex, race, ethnicity, insurance type, and duration of disease, baseline values of TIR and GMI that were farther from goal were associated with a greater improvement (*P*=.02 and *P*<.001, respectively). In addition, pump use was found to be associated with improvement in TIR, but not GMI (*P*=.045 and *P*=.36, respectively). Site, age, sex, race, ethnicity, insurance type, and duration of disease were not statistically associated with CGM metrics.

Screening laboratory tests were completed following 81/111 telemedicine visits (73%) compared with 138/157 visits in-person (87.9%) with 1 patient seen in-person lost to follow-up. This difference was statistically significant with patients seen in-person being 2.69 times more likely to have up-to-date screening laboratory tests by the next visit compared with those seen by telemedicine (*P*=.03). Diabetic emergencies could not be assessed following 9 visits where patients were lost to follow-up at the time of the data collection. However, there were only 2 instances (0.8%) of documented interim diabetic emergencies, and so were deemed too infrequent to meaningfully analyze.

**Table 1:** Patient demographics.

Characteristics		UCLA^a^ (n=39)	MCH^b^ (n=96)	Overall (n=135)
Sex, male, n (%)		19 (48.7)	54 (56.3)	73 (54.1)
Age (years), mean		13.5	13.2	13.3
Duration (years), mean		5.1	5.0	5.0
Private insurance, n (%)		36 (92.3)	43 (44.8)	79 (58.5)
Pump use, n (%)		23 (59)	52 (54.2)	75 (55.6)
**Race or ethnicity, n (%)**				
	White	29 (74.4)	25 (26.0)	54 (40.0)
	Hispanic or Latino^c^	1 (2.6)	57 (59.4)	58 (43.0)
	Black	0 (0)	7 (7.3)	7 (5.2)
	Asian	2 (5.1)	3 (3.1)	5 (3.7)
	Native Hawaiian	0 (0)	1 (1.0)	1 (0.7)
	American Indian	1 (2.6)	0 (0)	1 (0.7)
	Other	2 (5.1)	7 (7.3)	9 (6.7)
	Unknown	5 (12.8)	3 (3.1)	8 (5.9)

^a^UCLA: University of California, Los Angeles.

^b^MCH: Miller Children’s and Women’s Hospital.

^c^In the EHR, Hispanic or Latino ethnicity is coded separately and lists race as “other.”

## Discussion

### Principal Findings

Our study demonstrated a statistically significant improvement in both TIR and GMI at 2 and 4 weeks after outpatient type 1 diabetes visits compared with baseline values during 4 weeks before. This improvement was observed even after adjusting for patient demographics and study site and was similar by visit modality, indicating that clinically stable pediatric patients using CGM from diverse backgrounds can have immediate improvement in glycemic control following both telemedicine and in-person visits. While not statistically significant, there were greater improvements in TIR and GMI in the immediate 2-week postvisit period, with a slight reversal toward baseline at the 4-week period. Possible explanations include recency of reviewed diabetes education at the outpatient visit (ie, decreased retention of information over time), or potential decrease in motivation to follow recommendations as more time elapsed following a clinical encounter. This would be expected to be more prominent for patients on multiple daily injections since with insulin pump therapy, settings are programmed directly into the pump which alleviates some patient burden for remembering and incorporating dose adjustments. This may result in a greater likelihood for the patients on pumps to be compliant with current insulin regimen recommendations. Regardless, an overall improvement in both TIR and GMI was maintained at 4-weeks post visit, though the magnitude of these changes was small. Goal TIR is typically at least 70% and extrapolating GMI to HbA_1c_ targets suggest an ideal value of <7% (53 mmol/mol) for most pediatric patients [2,11]. This reinforces a need for multiple outpatient visits or insulin regimen adjustments over time to attain a clinically relevant, maintained change in TIR or GMI.

We found no significant difference in improvements in our CGM parameters of interest between patients seen in-person versus by telemedicine. Previous studies have shown that telemedicine is not inferior to in-person visits in terms of key diabetes clinical outcomes among patients on CGM [16]. A likely reason is that data provided by CGM can be easily generated and transmitted remotely, and thus, easily incorporated into a telemedicine type 1 diabetes outpatient visit. Therefore, when CGM data are available, the option for telemedicine encounters in patients who are clinically stable can be a viable alternative to traditional in-person assessment.

Completion of screening laboratory tests was significantly higher among patients seen in-person compared with those seen through telemedicine. This may be explained by the accessibility of on-site laboratory tests available to patients seen in-person (ie, blood samples in the laboratory can be conveniently collected after the visit). Patients seen in-person at UCLA always had access to an on-site laboratory, while only patients with public insurance seen at MCH could have blood drawn in the laboratory on site due to insurance contracts. Because not all pediatric endocrinology practices have access to an on-site laboratory, our findings may not be wholly generalizable. Two recent articles identified a similar pattern in adults, both reporting that patients had statistically higher completion of recommended laboratory tests if seen in-person versus by telemedicine [20,21]. These findings suggest that some degree of in-person visits remain beneficial to facilitate the completion of screening laboratory tests, and so perhaps a hybrid approach of using both telemedicine and periodic in-person evaluations would be appropriate.

Diabetic emergencies occurred too infrequently to meaningfully analyze in our data set (episodes occurred following 0.8% of visits). Certainly, this is influenced by the inclusion criteria of last HbA_1c_ <10% as poorly controlled patients are at much higher risk for hyperglycemia-related emergency department visits and hospitalizations. Another possible explanation could be the previously reported protective nature of CGM use. This was reiterated by a recent publication from the type 1 diabetes exchange data showing that CGM users were about one third as likely to endure a diabetic ketoacidosis event compared with nonusers [22].

Baseline TIR and GMI values farther from goal were associated with greater magnitude of change. This is not surprising as these patients inherently have more room for improvement. Also, while maximizing TIR is an appropriate goal in all patients, minimizing GMI in those with near optimal control can come at the cost of more frequent hypoglycemic episodes. However, we cannot exclude that a regression to the mean may also contribute to the bigger improvement in those with worse baselines. Pump use was also associated with greater improvement in TIR from baseline, although not in GMI. This implies that patients using insulin pumps had more blood sugars centered within the target range, while not necessarily affecting average blood sugar. This decreased variability has been historically reported in patients on insulin pumps, and intuitively makes sense if using a hybrid-closed loop algorithm that acts to mitigate both hypo- and hyperglycemia [23].

Our study design had several key strengths including a diverse patient population across both centers representing a spectrum of pediatric ages, durations of disease, and insurance types. Furthermore, within the study period many patients were seen by both telemedicine and in-person visits which mitigated potential patient demographic differences when comparing visit modality. However, there are several notable limitations of this study. This was a retrospective analysis of EHR data so our findings are subject to confounding factors that may have contributed to the patient or health care team decisions on whether to perform an in-person or telehealth visit. In addition, our results are limited to patients who met inclusion criteria of using Dexcom CGM with most recent HbA_1c_ <10%, and therefore may not be generalizable to children and adolescents using other CGM devices or traditional finger sticks for glucose monitoring, with lower CGM adherence rates, or with higher HbA_1c_ levels. Furthermore, there is potential for sampling bias since patients already using CGM to manage diabetes may have increased motivation or investment in their care to follow recommendations from outpatient encounters which could manifest as improved clinical outcomes. In addition, since rates of completion of screening laboratory tests were measured by being up to date by the following visit, it includes visits in which patients were not due for any screening laboratory tests and so it is possible that our 2 cohorts already had a baseline difference in the necessity for screening laboratory testing. Finally, our data are limited to 269 total patient encounters and therefore, larger scale and prospective trials are needed to verify our findings as well as to adequately analyze the risks of low frequency events such as diabetes emergencies.

### Conclusion

Our study found small but statistically significant improvements in TIR and GMI within 2-4 weeks following outpatient pediatric type 1 diabetes visits, which was not statistically different whether conducted in-person or through telemedicine. Because of the small magnitude of these changes, subsequent visits and CGM reviews remain important to achieve clinically relevant improvements over time. However, our study found that completion of screening laboratory tests occurred more often following in-person visits which highlights the importance of alternating between periodic telemedicine and in-person visits at a frequency according to individual patient care needs. Since multiple outpatient visits are necessary for a clinically meaningful and maintained impact on glycemic control, hybrid approaches using both telemedicine and in-person visits for type 1 diabetes care in pediatric patients to improve access to care and visit efficiency is an area in need of further study.

## Abbreviations

CGM: continuous glucose monitor
EHR: electronic health record
GMI: glucose management indicator
HbA1c: hemoglobin A1c
ICD-10: International Statistical Classification of Diseases, Tenth Revision
MCH: Miller Children’s and Women’s Hospital
TIR: time in range
UCLA: University of California, Los Angeles
